# Supplementary material for: Predicting gene level sensitivity to JAK-STAT signaling perturbation using a mechanistic-to-machine learning framework
Source: bioRxiv. 2023 May 20:2023.05.19.541151. Preprint. [Version 1] doi: 10.1101/2023.05.19.541151 (PMC10245690; doi:10.1101/2023.05.19.541151)
Supplement: Supplement 3 [file NIHPP2023.05.19.541151v1-supplement-3.pdf]

Fig. S1

bioRxiv preprint doi: https://doi.org/10.1101/2023.05.19.541151; this version posted May 20, 2023. The copyright holder for this preprint (which was not certified by peer review) is the author/funder, who has granted bioRxiv a license to display the preprint in perpetuity. It is made available under aCC-BY 4.0 International license.

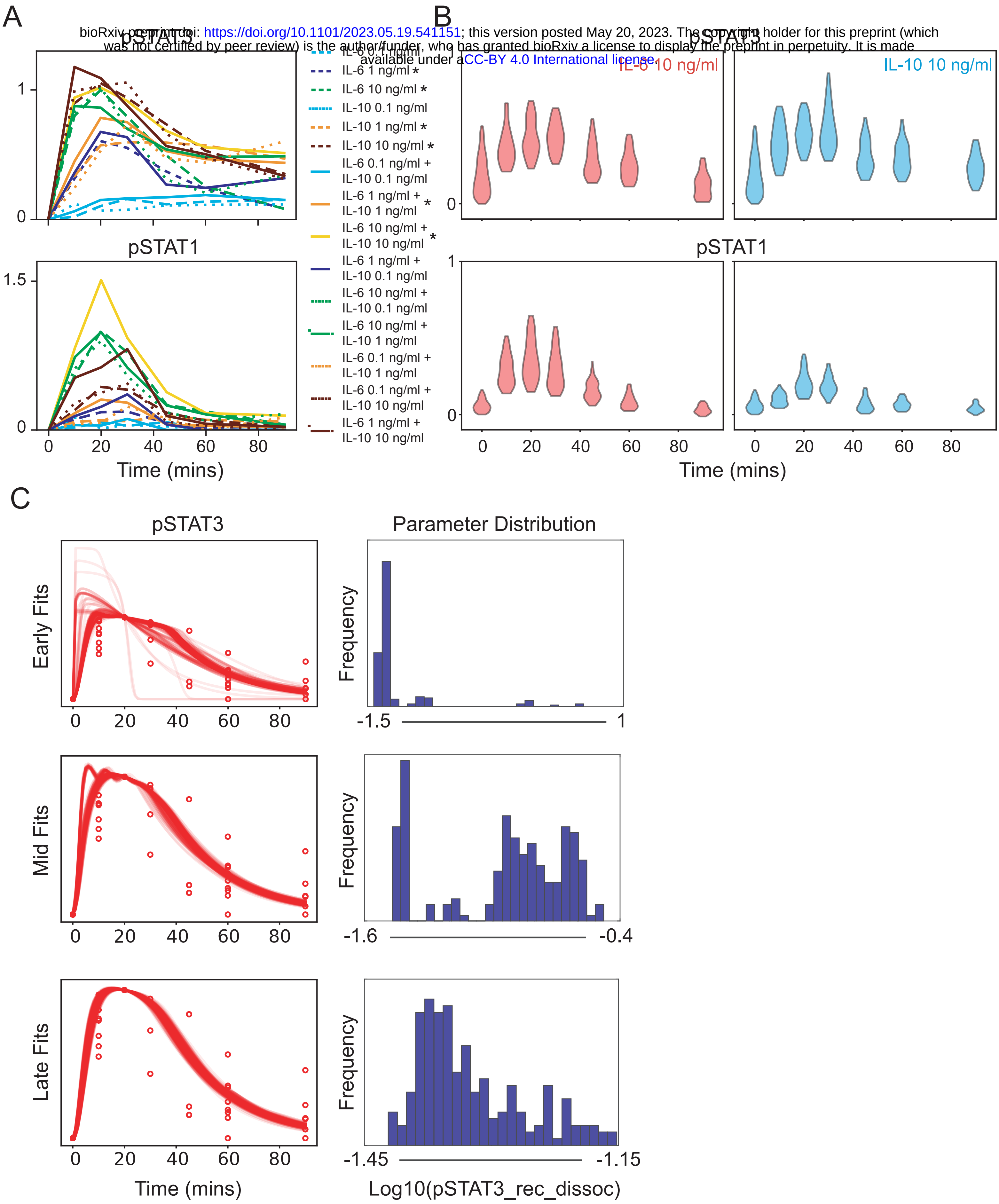

Fig. S2

bioRxiv preprint doi: https://doi.org/10.1101/2023.05.19.541151; this version posted May 20, 2023. The copyright holder for this preprint (which was not certified by peer review) is the author/funder, who has granted bioRxiv a license to display the preprint in perpetuity. It is made available under aCC-BY 4.0 International license.

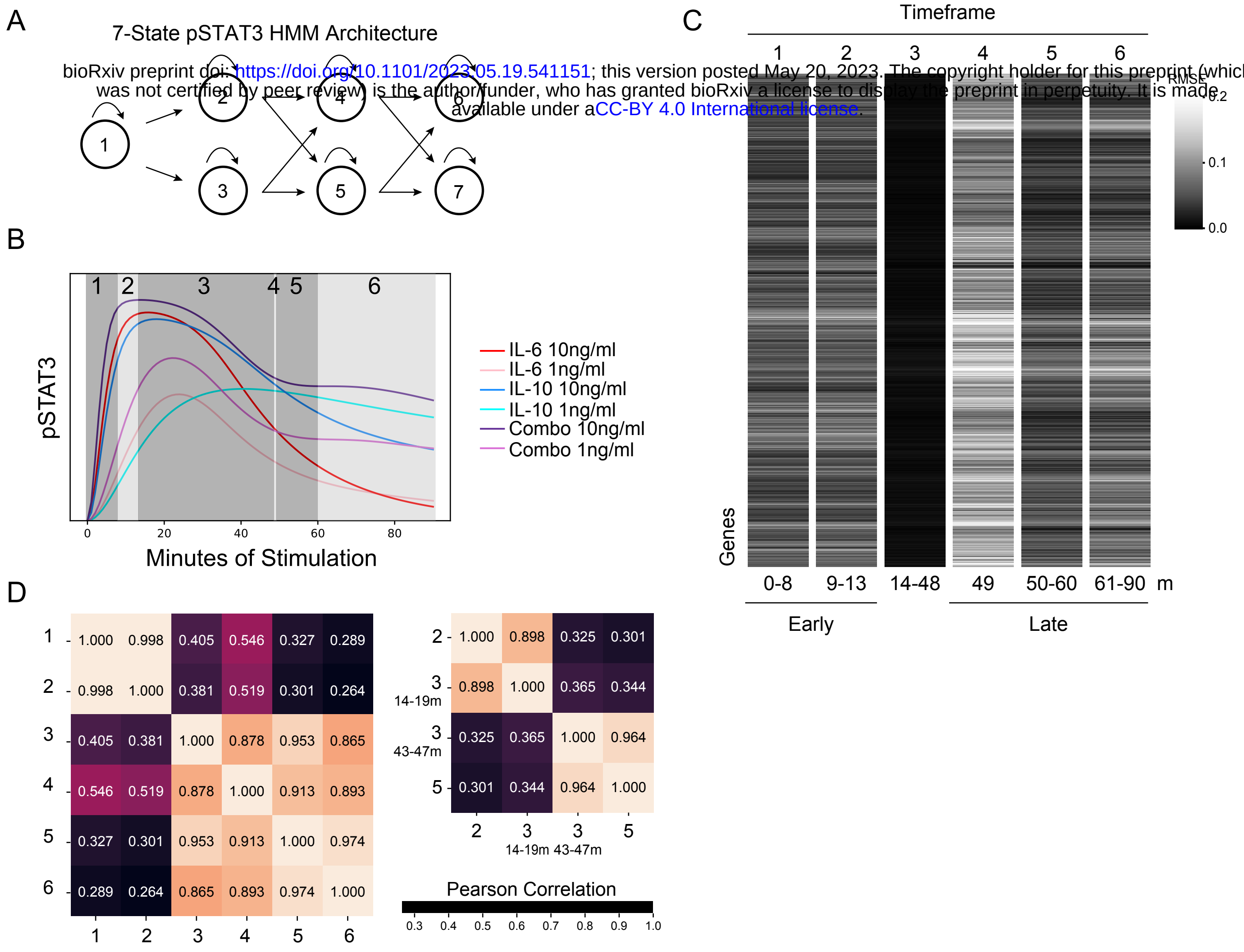

Fig. S3

bioRxiv preprint doi: https://doi.org/10.1101/2023.05.19.541151; this version posted May 20, 2023. The copyright holder for this preprint (which was not certified by peer review) is the author/funder, who has granted bioRxiv a license to display the preprint in perpetuity. It is made available under aCC-BY 4.0 International license.

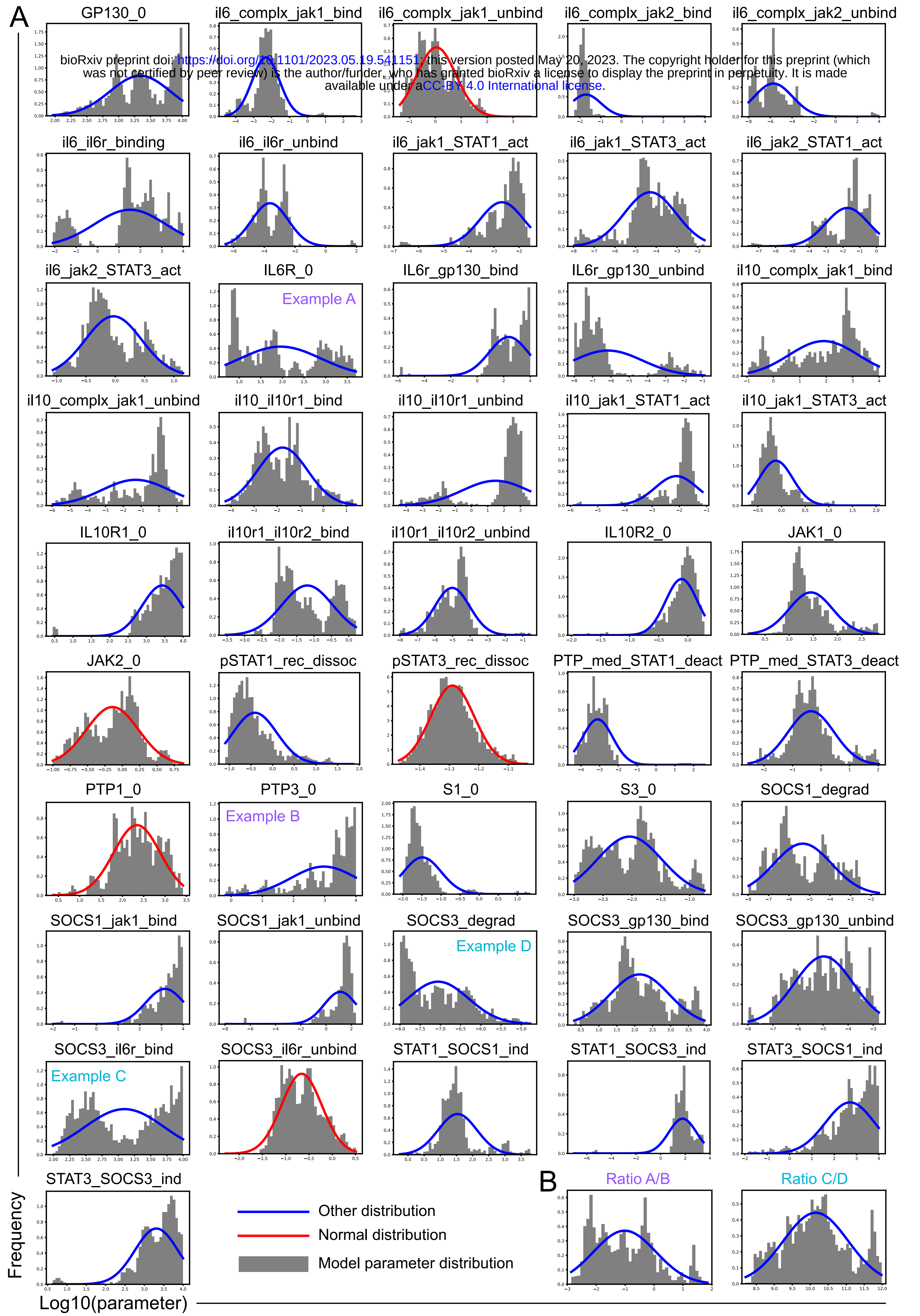

Fig. S4

bioRxiv preprint doi: https://doi.org/10.1101/2023.05.19.541151; this version posted May 20, 2023. The copyright holder for this preprint (which was not certified by peer review) is the author/funder, who has granted bioRxiv a license to display the preprint in perpetuity. It is made available under aCC-BY 4.0 International license.

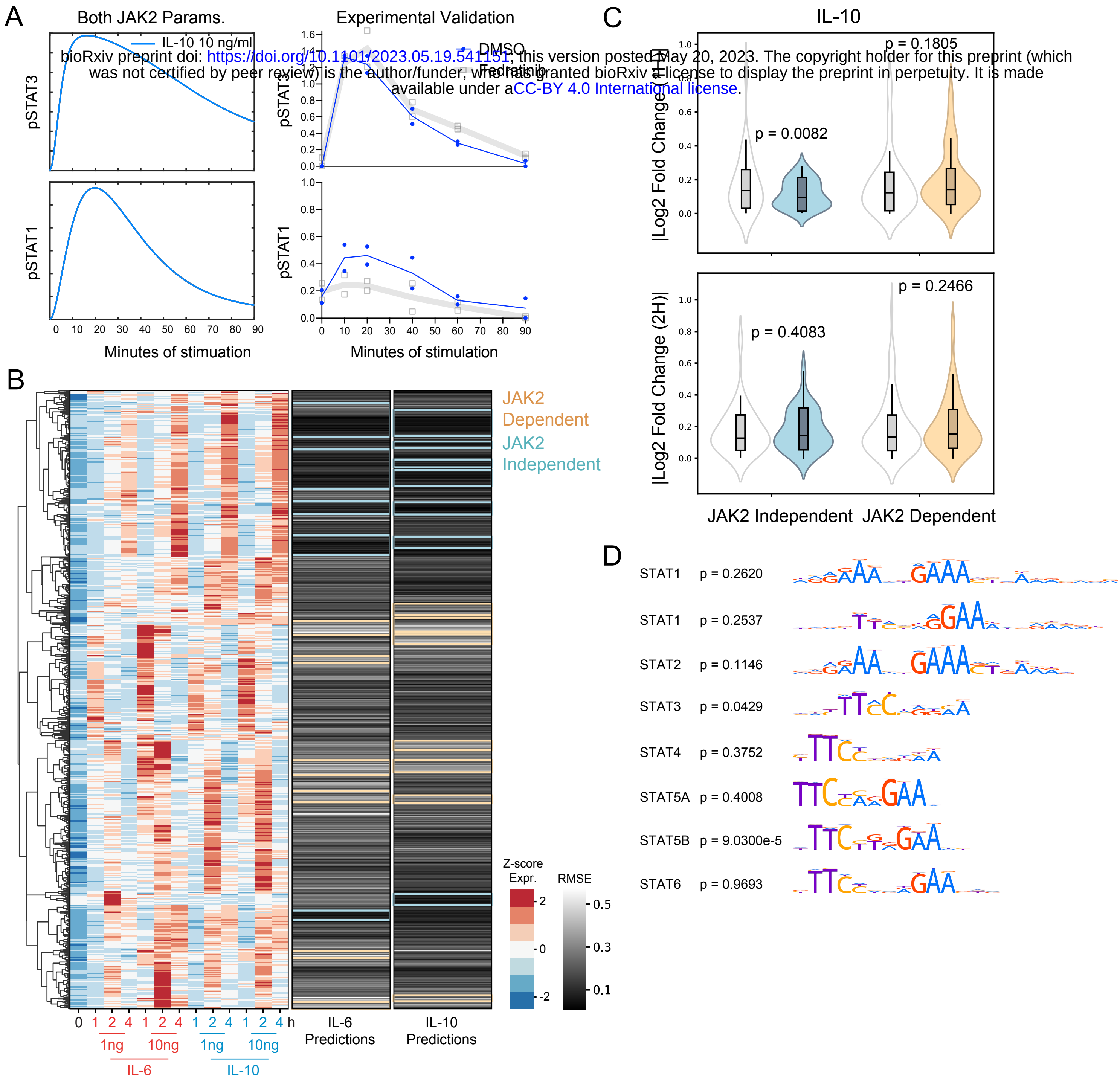

bioRxiv preprint doi: https://doi.org/10.1101/2023.05.19.541151; this version posted May 20, 2023. The copyright holder for this preprint (which was not certified by peer review) is the author/funder, who has granted bioRxiv a license to display the preprint in perpetuity. It is made available under aCC-BY 4.0 International license.

Figure S1. Detailed analysis of pSTAT dynamics and model fitting steps. (A) Cytokine-induced pSTAT dynamic curves colored according to K-means clustering results. Asterisks note conditions selected for RNAseq and all further analysis. (B) Violin plots of a representative experiment showing single-cell distributions of IL-6 or IL-10-induced (10 ng/ml) pSTAT1 and pSTAT3. (C) Parameter estimation results at early, middle (mid), and late stages of fitting process (left column) with corresponding Log_{10} of selected model parameter (right column). Detailed description of parameter estimation process in Methods.

Figure S2. Early versus late STAT3 dynamic timeframes identified using a Hidden Markov Model (HMM). (A) 7-state HMM architecture used to identify dynamic STAT3 phosphorylation states (hidden states). Gaussian emission HMM was trained with the Baum-Welch algorithm on 10,000 bootstrapped runs with 250 model-simulated, cytokine-induced pSTAT3 samples each, and the transition matrix, means, and covariances of the states were averaged across runs for the final HMM. The HMM hidden states were decoded in test pSTAT3 trajectories using the Viterbi algorithm. (B) 6 pSTAT3 timeframes defined by HMM. Boundaries of timeframes were identified by selecting the time points at which each trajectory underwent a transition to a new state (from Viterbi decoding), and those time points were averaged across all cytokine-induced trajectories to be used as timeframe boundaries. The overlapping portions of timeframes were considered their own timeframe. (C) Gene expression prediction errors (RMSE) averaged across time points per timeframe using the prediction pipeline in Fig. 3A. (D) Correlation matrices of timeframe gene predictions, demonstrate that timeframes 1 and 2 exhibit similar prediction behaviors, while timeframes 3 through 6 exhibit similar prediction behaviors (left matrix). Correlations of the early and late portion of timeframe 3 suggest that it contains dynamic information related to the early and late responses (right matrix). Timeframes 1 and 2 correspond to “early” in Figure 3, while timeframes 5 and 6 correspond to “late”.

Figure S3. Rule-based model parameters are under constrained by experimental data. (A) Distributions of Log_{10} model parameter values. Colors represent normality testing results from the Shapiro-Wilk test, with red curves showing distributions deemed log normally distributed in more than half of bootstrapped runs. (B) Parameter ratio distributions of randomly selected unrelated model parameter pairs, for comparison to correlated parameter ratios in Figure 4B.

Figure S4. Model dynamic and gene expression predictions of IL-10-induced JAK2 inhibition are limited. (A) Impact of up to 3-fold decrease (grayscale) in both JAK2 protein levels

bioRxiv preprint doi: https://doi.org/10.1101/2023.05.19.541151; this version posted May 20, 2023. The copyright holder for this preprint (which was not certified by peer review) is the author/funder, who has granted bioRxiv a license to display the preprint in perpetuity. It is made available under aCC-BY 4.0 International license.

and IL-6-induced JAK2 activation of STAT1 on IL-10-induced (10 ng/ml) pSTAT model trajectories (baseline in blue). The grayscale curves are obscured by the original blue trajectory because JAK2 does not impact the simulated IL-10-induced response. Data on the right show pSTAT IF data of IL-10-stimulated (10 ng/ml) BMDM with and without 1 nM Fedratinib treatment (n = 2). (B) Hierarchical clustering of upregulated, z-score normalized DEGs across pooled, single cytokine samples (n = 2), average gene expression prediction errors across the 1 and 2 hour, for IL-6-induced and IL-10-induced pSTAT1 gene predictions. Groups of predicted JAK2-dependent and predicted JAK2-independent genes that meet the 90th and 10th gene expression percentile cutoffs, respectively, noted by the colored boxes (see Methods for cluster identification). (C) Absolute value of Log_2 fold change for IL-10 + Fedratinib treated with respected IL-10 only; predicted JAK2-dependent and predicted JAK2-independent genes were compared to randomly selected genes. P-values were determined using one-sided Welch's t-test. D) Adjusted p-values and motif logos for enrichment of canonical STAT motifs in predicted JAK2-dependent versus predicted JAK2-independent genes.

Table S1. Table S1. Rule-based model parameter names and descriptions.

Supplemental File 1. Genes in late pSTAT3 predicted clusters noted by asterisks in Fig. 3C.
